# Parents willingness to vaccinate their daughter against human papilloma virus and its associated factors in Bench-Sheko zone, southwest Ethiopia

**DOI:** 10.1016/j.heliyon.2021.e07051

**Published:** 2021-05-17

**Authors:** Alemnew Destaw, Tewodros Yosef, Biruk Bogale

**Affiliations:** Department of Epidemiology and Biostatistics, School of Public Health, College of Medicine and Health Sciences, Mizan Tepi University, Mizan Aman, Ethiopia

**Keywords:** Parental willingness, HPV vaccine, Ethiopia

## Abstract

**Background:**

In Ethiopia, the human papillomavirus vaccine has been introduced since 2018. Since the vaccination program targets girls age 9–13, the success of vaccination depends on the parental decision and their willingness to vaccinate their daughters. Therefore, a study on parental willingness to vaccinate their daughter and factors associated is needed.

**Objective:**

To assess parent's willingness to vaccinate their daughter against the human papillomavirus and its associated factors in Bench-Sheko Zone, southwest Ethiopia.

**Methods:**

A community-based cross-sectional study was conducted among 502 participants in Bench-Sheko Zone, southwest Ethiopia. The participants were selected using a systematic random sampling method. Frequency tables, mean and standard deviation were used to summarize the data. A binary logistic regression using bivariate and multivariable logistic regression analysis was used to identify factors associated with parental willingness to vaccinate their daughter. The level of significance was declared at P-value < 0.05.

**Results:**

Of the 502 participants interviewed, 399 (79.5%), 95% CI (76%, 83%) of parents were willing to vaccinate their daughter. The study found that primary education and above (AOR = 2.9, 95% CI [1.79, 4.95]), having good knowledge (AOR = 2.1, 95% CI [1.15, 4.10]) and positive attitude (AOR = 2, 95% CI [1.30, 3.41]) were significantly associated with parental willingness to vaccinate their daughter.

**Conclusion:**

This study found that there was a high parental willingness to vaccinate their daughter against the human papillomavirus in the study area. Primary education and above, having good knowledge and positive attitude were factors associated with parental willingness to vaccinate their daughter. Therefore, providing health information's regarding human papillomavirus vaccination with emphasis to raise community awareness should be designed especially less educated parents need to be targeted.

## 1 Introduction

Cervical cancer is the fourth most common cancer diagnosed among women worldwide [1]. More than 80% of the cases are in developing countries [1]. In Ethiopia, cervical cancer becomes the second leading cause of cancer death [1]. Cervical cancer prevention programs such as HPV vaccination and cytology-based cervical cancer screening programs decreased cervical cancer incidence in developed countries [2]. However, in developing countries, lack of access to effective screening and low screening coverage, poverty, low education, and life in rural areas [3, 4] coupled with a high incidence of HPV 16 and 18 infections [5] increases cervical cancer incidence.

Persistent human papillomavirus (HPV) infection, a sexually transmitted disease, is identified as the necessary cause of cervical cancer particularly HPV 16, 18, and 31 serotypes [6, 7]. More than 80% of sexually active women are infected by HPV at least once during their lifetime [8]. In response to this, the world health organization develops comprehensive strategies for the prevention and control of cervical cancer including HPV vaccination as the primary prevention method for cervical cancer [9]. Three HPV vaccines namely bivalent (Cervarix, GlaxoSmithKline), quadrivalent (Gardasil, Merck), and nonavalent (Gardasil, Merck) vaccines are currently available, and have proven effective at protecting against HPV 16 and HPV 18 infections [10]. which are currently approved for vaccination in developing countries by WHO [11]. In Ethiopia, HPV vaccine with the support of the Global Alliance for Vaccine and Immunization (GAVI) has been introduced since 2018 being delivered through a school-based approach [12] as Ethiopia fulfilled the GAVI Alliances eligibility criteria for HPV vaccination support [13]. WHO recommends the vaccination to girls of age 9–13 years old [14].

Given the vaccination program targets adolescent girls, the success of vaccination depends on parental decisions and their willingness to vaccinate their daughters [15, 16] therefore it is needed to assess parental willingness to vaccinate their daughter in Ethiopia. A study in Gonder, Ethiopia found that 81.3% of parents accepted the HPV vaccine for their daughter [17] However it was conducted only in urban areas, and research in rural residents was also needed. Furthermore, previous studies were also conducted on knowledge, attitude, and vaccine acceptance among university students [12, 18] and among health professionals [19].

To the best of our knowledge, the study on parental willingness to vaccinate their daughters by including rural parents in Ethiopia and study area in particular not yet available. Therefore, this study aimed to assess parental willingness to vaccinate their daughter and its associated factors in Bench-Sheko zone, southwest Ethiopia.

## 2 Methods and materials

### 2.1 Study design, area, and period

A community-based cross-sectional study was conducted among residents in the Bench Sheko zone from December 20, 2020, to January 20, 2021. Bench-Sheko zone is found at 585 km southwest of Addis Ababa, the capital of Ethiopia. The Bench-Sheko zone is administratively divided into six woredas (districts) and two town administrations. The total population in the study area was projected to be 847,168 (417,751 males and 429,417 females) in 2017 [20].

### 2.2 Populations

**Source population:** All parents who had daughters age 9–13 years old in Bench-sheko zone.

**Study population:** Selected parents who had daughters age 9–13 years old in Bench-sheko zone during data collection time.

**Inclusion criteria:** Parents who had daughters age 9–13 years old and permanently residing in the study area (more than 6 months) were included in the study.

### 2.3 Sample size determination

The single population proportion formula was used to determine the required sample size for the study; considering the proportion of parental vaccine acceptance (P = 81.3%) from a previous study conducted in Gondar town Ethiopia [17], 95% confidence level, 5% margin of error, 10% non-response rate and a design effect of 2. The final computed sample size was 514.

### 2.4 Sampling technique and procedure

A multistage random sampling technique was applied. In the first stage, two woredas (Sheko and Shey Benchi) and Mizan- Aman town administrations were selected using the lottery method. Then, thirty percent of Kebeles (smallest administrative units) were selected from each of the selected woredas. Then, the calculated samples were distributed to each of the selected Kebeles using the proportional to sample size allocation (PCA) principle. Finally, the households were selected using a systematic random sampling technique with every 'k^th^’ skip interval. Consequently, households with parents who had daughters age 9–13 were interviewed.

### 2.5 Data collection technique and data quality control

A structured quantitative questionnaire was used for data collection developed after reviewing relevant works of literature [12, 17, 19]. The questionnaire is composed of three parts. Parts 1 assess the socio-demographic characteristics of the participants, part 2 contains knowledge assessment items, and part 3 contains attitude and willingness to HPV vaccine assessment questions. The questionnaire was translated into the local language (Amharic) and translated back to English to keep its consistency. Then, the tool was pre-tested on 5% of the sample size from Shishoinde kebele from which the actual study did not include. Five experienced data collectors and two supervisors were recruited and trained regarding the purpose of the study, how to collect the data, confidentiality and how to protect themselves from COVID-19 during data collection as per the Ethiopian ministry of health COVID-19 prevention guidelines [21]. The reliability of the tool was ensured by calculating the cronbaches alpha coefficient for likert scale items (0.82) and knowledge items (0.78).

### 2.6 Study variables and measurements

The dependent variable was parents’ willingness to vaccinate their daughter.

The independent variables were socio-demographic factors (Age, sex, educational status, marital status, occupational status, and monthly income level), knowledge status, and attitude towards HPV vaccination.

**Knowledge:** Knowledge about HPV infection and its vaccination was assessed using 17 yes or no response items. A correct response was levelled as 1, otherwise 0. The scores for each items summed, and then those respondents who scored greater than the mean value was categorized as good knowledge, otherwise poor knowledge [22].

**Attitude:** Attitude was assessed using six likert scale items ranging from strongly disagree to strongly agree with a maximum score of 5 × 6 = 30. Those who scored greater than the mean value was categorized as having a positive attitude, otherwise negative attitude [17].

**Willingness to the HPV vaccine:** Parents who respond ‘yes’ to the question ‘will you vaccinate your daughter for HPV vaccine’ considered as ‘willing’ otherwise ‘not-willing’ [17, 22].

### 2.7 Data management and analysis

The data were entered into Epi data manager version 4.0.2.101 and analysed using SPSS version 21. The data were presented using frequency tables, and numerical summary measures (mean and standard deviation). A binary logistic regression analysis was used to assess the association between the independent variables with the dependent variable. Crude odds ratio (COR) and adjusted odds ratio (AOR) with its respective 95% confidence interval (CI) was used to interpret the result. A Multi-collinearity test between independent variables was checked using the Variance inflation factor and correlations were tolerable (VIF<2). Model goodness of fit test was cheeked using Hosmer- Lemeshow test and it was a good fitted model (P-value = 0.20). The independent variables with p-values <0.25 in the bivariate analysis were candidate variables for multivariable logistic regression analysis. The level of significance was declared at a P-value <0.05.

### 2.8 Ethical consideration

Ethical approval was obtained from Mizan-Tepi University College of health science research ethics committee (Reference number/SPH/0017/2021/) and a letter of cooperation were granted from the study area. Written and verbal informed consent was taken from all study participants.

## 3 Result

### 3.1 Socio-demographic characteristics of the participants

Of the 514 parents contacted by data collectors to participate in the study, 502 participants were interviewed yielding a response rate of 97.6%. The mean age of respondents was 28.6 (±6) years old. The majority (69.5%) of the respondents were in the age group of under 30 years old, and 378 (75.3%) were mothers. More than half (55%) of participants were rural residents (Table 1).

**Table 1:** Distribution of willingness to vaccinate according to socio-demographic characteristics, knowledge and attitude of participants in Bench-sheko zone, 2021.

Variables	Categories	Total (n = 502)	Willingness	
			No (20.5%)	Yes (79.5%)
Age group	<30	349 (69.5)	75 (73.0)	274 (68.7)
	≥30	153 (30.5)	28 (27.0)	125 (31.3)
Sex	Female	378 (75.3)	79 (76.7)	299 (74.9)
	Male	124 (24.7)	24 (23.3)	100 (25.1)
Residence	Urban	226 (45.0)	38 (36.9)	188 (47.1)
	Rural	276 (55.0)	65 (63.1)	211 (52.9)
Educational status	Non-educated	220 (43.8)	68 (66.0)	151 (37.8)
	Primary edu& above	282 (56.2)	35 (34.0)	248 (62.2)
Monthly income	<42$	132 (26.3)	24 (23.4)	108 (27.1)
	42$-85$	140 (27.9)	39 (37.8)	101 (25.3)
	>85$	230 (45.8)	40 (38.8)	190 (47.6)
Knowledge status	Poor	360 (71.7)	89 (86.4)	271 (67.9)
	Good	142 (28.3)	14 (13.6)	128 (32.1)
Attitude	Negative	154 (30.7)	49 (47.6)	105 (26.3)
	Positive	348 (69.3)	54 (52.4)	294 (73.7)

### 3.2 Parental willingness for HPV vaccination

Of the 502 parents interviewed, 399 (79.5%), 95% CI (76%, 83%) of them were willing to vaccinate their daughter against HPV infection (Table 2).

**Table 2:** Bivariable and Multivariable logistic regression analysis for factors associated with parental willingness to vaccinate in Bench-sheko zone, 2021.

Variables	Category	Willingness		COR (95%CI)	AOR (95%CI)
		No (%) 103 (20.5%)	Yes (%) 399 (79.5%).		
Age group	<30	75	274	1	1
	≥30	28	125	1.2 (0.75,1.98)	1.6 (0.95,2.77)
Sex	Female	79	299	1 (0.66,1.83)	1 (0.62,1.88)
	Male	24	100	1	1
Residence	Urban	38	188	1.5 (0.97,2.38)	1.5 (0.96,2.49)
	Rural	65	211	1	1
Income level	<42$	24	108	0.9 (0.54,1.65)	1.6 (0.88,3.02)
	42$-85$	39	101	0.5 (0.33,0.90)	0.7 (0.40,1.19)
	>85$	40	190	1	1
Educational status	Illiterate	68	151	1	1
	Primary& above	35	248	3 (2.02,5.03)	2.9 (1.79,4.95)
Knowledge status	Poor	89	271	1	1
	Good	14	128	3 (1.64,5.47)	2.1 (1.15,4.10)
Attitude	Positive	49	105	2.5 (1.62,3.97)	2 (1.30,3.41)
	Negative	54	294	1	1

CI; Confidence interval; 1$ = 35ETB; COR = Crude odds ratio; AOR: Adjusted odds ratio.

### 3.3 Factors associated with the parental willingness for HPV vaccination

In the bivariate analysis residence, educational status, knowledge status, and attitude status were significantly associated with parental willingness to HPV vaccination. In multivariable logistic regression analysis; educational status, knowledge status, and attitude were significantly associated with parental willingness to HPV vaccination.

Those parents with primary and above education were more likely for HPV vaccination than those parents with no education AOR: 2.9, 95% 95% CI (1.79, 4.95). Those parents who had good knowledge about HPV and HPV vaccinations were more likely to HPV vaccinate their counterparts AOR: 2.1, 95% CI (1.15, 4.10). Those parents with a positive attitude about HPV and HPV vaccination were more likely to be willing to HPV vaccinate their children than their counterparts AOR: 2, 95% CI (1.30, 3.41) (Table 2). Despite age group, residence and income showed association (AOR>1), these variables had no statistically significant association with parental willingness to HPV vaccination.

## 4 Discussion

This study found that there was a high parental willingness to have their daughter vaccinated for HPV. Primary and above education, having good knowledge and a positive attitude were factors associated with parental willingness.

In this study more than two-third of parents were willing for HPV vaccination 399 (79.5%). The finding of this study is consistent with other studies; in Mali, Kenya, Argentina, and Nigeria [23,24,25,26], and Ethiopia [17]. A study in Gondar showed that 81.3% of parents were accepted the vaccine for their daughter [17] similarly in Mali 74.5% of parents were willing to vaccinate their daughter [23] 81.8% in Nigeria in 2017 [26]. This suggests that the high parental willingness to vaccinate their daughter in Ethiopia is positive news and a good opportunity as well given the country has introduced a school-based vaccination campaign to these targeted girls. A more willing parent to get their daughter vaccinated for HPV will ease the vaccination campaign by having more girls available for vaccination as parents have an invaluable part in a decision to get or not the vaccine by targeted girls.

In contrast to this, the current finding is lower than some studies that reported willingness to HPV vaccination for their daughter as 89% in Kenya in 2015 [22]. The difference might be due to the difference in the source population used by these studies. The source population (Kenyan study) was school teachers unlike parents in the current study. School teachers might have a better understanding of HPV infection due to their high awareness about HPV vaccination (was 90% in that study) which might put them for their high willingness to vaccinate their daughters than our study.

In this study, those parents with primary education and above were more likely willing to vaccinate their daughter than those with no formal education. This is in line with other studies that reported similar findings [26, 27]. This suggests that the educational level of parents plays a significant role in their acceptance of the vaccine campaign for their daughters. Therefore specific educational programs about HPV vaccination need to be designed and targeted for those parents with no formal education to increase their willingness towards the vaccination program in doing so will increase vaccine attendance by their daughters in the future. However apart from parental educational status, there was no significant association between other socio-demographic characteristics of parents and willingness to vaccinate their daughter. This is supported by previous study which reported similar finding [28].

In this study, one in three parents had good knowledge about human papillomavirus. Good knowledge was also a significant factor for high parental willingness to vaccinate their daughter in the study area. This is consistent with other studies conducted in Gondar town Ethiopia and Kenya [17, 22] this highlights parental willingness to vaccinate their daughter is affected by their overall knowledge about the HPV vaccine. Therefore parental awareness raising programs is a key and need to be designed specifically for parents with no formal educations.

Parental positive attitude towards HPV vaccine was significantly associated with parental willingness to vaccinate their daughter. This is supported by other study findings [17, 29, 30]. This highlights that regardless of poor knowledge level by parents, more than two-thirds of parents had a positive attitude about the HPV vaccine and if vaccination service is available, they would be willing to vaccinate their daughter. Therefore educational information designed for parents with a negative attitude to HPV vaccine would result from high vaccine acceptance thereby increases vaccine attendance in the future.

### 4.1 Strength and limitations

A community-based study using a representative sample that includes parents from the rural area of Ethiopia could be possible to show the current parental willingness to vaccinate their daughter to the vaccine campaign currently implemented by Ethiopian MOH and Bench Sheko zonal health Department. Our study finding was based on the previous WHO recommendations for HPV vaccination, and this might not reflect the updated recommendation for vaccination to girls’ aged 9–14 years so that the finding should be interpreted cautiously. Since the study is a cross-sectional study, temporal association of factors with the dependent variable could not be possible.

## 5 Conclusion and recommendations

This study found that there was a high parental willingness of HPV vaccination to vaccinate their daughter in the study area. Primary education and above, having good knowledge and positive attitude were factors associated with parental willingness to vaccinate their daughter. The Ethiopian ministry of health, regional and zonally health departments should design health information's regarding HPV vaccination with emphasis to raise community awareness especially to those less educated parents.

## Declarations

### Author contribution statement

Alemnew Destaw: Conceived and designed the experiments; Performed the experiments; Analysed and interpreted the data; Wrote the paper.

Tewodros Yosef and Biruk Bogale: Performed the experiments; Analysed and interpreted the data; Contributed reagents, materials, analysis tools or data; Wrote the paper.

### Funding statement

This work was supported by Mizan Tepi University College of Health Science.

### Data availability statement

Data will be made available on request.

### Declaration of interests statement

The authors declare no conflict of interest.

### Additional information

No additional information is available for this paper.
